# Nutritional, Biochemical, and Functional Properties of Spinach Leaf-Enriched Dough: A Healthier Alternative to Conventional Pasta

**DOI:** 10.3390/foods13223608

**Published:** 2024-11-12

**Authors:** Ilaria Iacobellis, Alessia Lisi, Mirco Vacca, Carmen Aurora Apa, Giuseppe Celano, Leonardo Mancini, Fabio Minervini, Maria Calasso, Maria De Angelis

**Affiliations:** Department of Soil, Plant and Food Science, University of Bari Aldo Moro, via G. Amendola 165A, 70126 Bari, Italy; ilaria.iacobellis@uniba.it (I.I.); alessia.lisi@uniba.it (A.L.); carmen.apa@uniba.it (C.A.A.); giuseppe.celano@uniba.it (G.C.); leonardo.mancini1@uniba.it (L.M.); fabio.minervini@uniba.it (F.M.); maria.deangelis@uniba.it (M.D.A.)

**Keywords:** spinach pasta, pasta fortification, source of fiber, food perception, functional food, consumer health

## Abstract

This study explored the effects of spinach flour (SF) enrichment on pasta, focusing on chemical, nutritional and sensory properties, cooking performance, and microbiological stability. SF was added at 12.5% (PSP12) and 25% (PSP25). The enriched pasta had a lower pH than the control (CP), due to spinach-derived organic acids, with PSP25 showing the highest fiber content. Enrichment increased B vitamins and minerals, especially calcium, magnesium, sodium, and potassium. PSP25 had a shorter cooking time, higher water absorption, and greater cooking loss. Enriched pasta showed lower starch hydrolysis index and predicted glycemic index, suggesting potential benefits for managing postprandial blood sugar levels. SF significantly altered the free amino acid (FAA) profile, with PSP25 showing the highest concentration of total FAAs. Antioxidant assays demonstrated that spinach-enriched pasta retained higher levels of phenols and flavonoids, after cooking also, compared to CP. Sensory analysis indicated that while PSP12 had higher overall acceptability, PSP25 exhibited stronger herbaceous flavors, which could affect consumer preference. Microbiologically, all samples were stable for 110 days. The findings suggest that SF enrichment enhances the nutritional value, antioxidant potential, and sensory qualities of pasta, with potential for commercial applications, although consumer acceptance could be influenced by its non-traditional taste and texture.

## 1. Introduction

Traditional pasta consists of a non-fermented dough comprising a mixture of only two ingredients, durum wheat semolina and water. One of the best features of pasta, contributing to its large application, is the opportunity to store it for extended periods at room temperature without losing its nutritional value or flavor. Pasta is also quick, easy, and versatile to prepare due to its short cooking time. According to United States Department of Agriculture (USDA), carbohydrates should make up about 45–65% of total daily caloric intake [1]. Thus, considering pasta as a primary carbohydrate source, it offers a significant contribution to the daily intake of this class of macronutrient, together with vitamins (mainly folic acid) and minerals (in particular selenium, copper, magnesium, and zinc) [2,3]. Durum wheat is the primary raw material used in this preparation, and its characteristics, including a high protein content, notably affect the dough’s rheological properties and cooking performance, defining, therefore, its final quality attributes [4]. However, the demand for products with high health benefits—particularly those rich in antioxidants, high in total polyphenols and fiber, low in fat, and produced with minimal environmental impact—has influenced recent trends in food fortification. Hence, according to its simple dough constituents, pasta has been shown to be ideal for incorporating additional ingredients, paving the way to easily develop novel functional foods [5]. Driven by consumer needs and preferences, the pasta industry has undertaken significant changes to enhance the nutritional profile and health benefits of this staple food, meeting the market demand for a healthier and more sustainable diet [6]. Various alternative ingredients, such as non-wheat grains, legumes, vegetables, and resistant starches, have been explored to partially replace wheat and improve quality attributes [7,8], and these studies showed an overall improvement in the chemical properties of the new formulations and an enhanced visual appeal of the pasta, but evidence for cooking loss and pasta firmness were not always favorable, varying with the amount of ingredient used. Enriching semolina pasta with additional ingredients allows the improvement of the chemical profile, enhancing the appeal [9] and providing bioactive compounds with radical scavenging properties, including phenolic compounds, vitamins, alkaloids, and other phytochemicals that positively affect human health and may lower the risk of numerous diseases [10,11]. Epidemiological studies suggested that the long-term consumption of polyphenol-rich foods can protect against various diseases, such as cancers, diabetes, osteoporosis, and neurodegenerative and cardiovascular disease [12]. Moreover, the types of foods consumed have a profound effect on the composition and diversity of gut microbiota. For example, diets rich in fiber, plant-based foods, and fermented products promote the growth of beneficial bacteria such as bifidobacteria and lactobacilli [13], and different nutrients are metabolized by gut bacteria to produce metabolites that impact, positively or negatively, health [14]. For instance, the fermentation of dietary fibers by gut microbiota results in the production of short-chain fatty acids (SCFAs) like butyrate, propionate, and acetate, which have anti-inflammatory properties and play roles in gut health and metabolism [15]. Polyphenols, including phenolic acids and flavonoids, are the most abundant secondary metabolites in different plants, and have been shown to benefit human health directly [16] or through gut microbiota metabolism [17]. Therefore, increasing the phenolic compound content in the final product yields important health benefits [18]. However, pasta fortification also represents a way to provide a good daily source of proteins, fibers, vitamins and other micronutrients that, according to EC Reg. 1924/2006 [19], can be identified in products based on their specific molecule concentration.

Spinach (*Spinacia oleracea*) is a highly nutritious vegetable that offers numerous health benefits. Its rich content of vitamins, minerals, and antioxidants contributes to overall well-being in various ways. One of the key advantages of spinach is its high levels of iron [20], which are essential for maintaining healthy blood and supporting oxygen transport throughout the body. In addition to iron, spinach is packed with vitamins A, C, and K, which help boost the immune system, improve skin health, and support bone strength [21]. The antioxidants found in spinach, such as lutein and zeaxanthin, are particularly beneficial for eye health. Furthermore, the anti-inflammatory properties of spinach can contribute to heart health by reducing inflammation in the arteries, thus lowering the risk of cardiovascular diseases [22]. Spinach is also low in calories and high in fiber, making it an excellent choice for those looking to maintain or lose weight. Its fiber content promotes healthy digestion and helps regulate blood sugar levels, making it a suitable option for individuals with diabetes [23]. Moreover, the presence of magnesium in spinach supports muscle function, nerve transmission, and energy production, which are all vital for maintaining a healthy body [24]. Overall, incorporating spinach into your diet can lead to improved energy levels, better digestion, stronger bones, and enhanced protection against chronic diseases, making it a valuable addition to a balanced and healthy lifestyle.

The aim of the present study was to investigate the beneficial effects of fortified pasta with the addition of flour obtained from spinach leaves. Samples were, at first, studied in terms of rheological parameters to obtain the best percentage of substitution of spinach flour and, according to panelist approval, for chemical composition, nutritional profiling, shelf-life assessment and presumptive functional activity in terms of scavenging activity, effects on gut microbiota, and fecal metabolome in vitro.

## 2. Materials and Methods

### 2.1. Ingredients

The spinach (*Spinacia oleracea*) flour (SF) was obtained by drying (6 h, 70 °C) and grinding (particle size < 200 μm) fresh spinach leaves. The nutritional facts, provided by the producer (Molino Bongiovanni, Villanova Mondovì, Italy), are detailed in Supplementary Table S1. Semolina was supplied by Industria Molitoria F.lli Martimucci S.r.l. (Altamura, Italy) and the nutritional contents were moisture 15%, carbohydrates 75%, fibers 3%, proteins 13.3%, dry gluten 11%, fats 1.6%, and ash 0.75%. The semolina particle size ranged 180–355 μm.

### 2.2. Preparation of Experimental Pasta

Spinach-containing pasta (SP) in the shape of “tagliatelle” was preliminarily produced on the laboratory scale at the University of Bari Aldo Moro using a manual pasta machine (Marcato Atlas 150 Pasta; Campodarsego (PD), Italy). Different percentages (3, 5, 10, 25%) of semolina substitution with SF were tested to evaluate the effects on the rheological properties of the dough, and to achieve desirable nutritional and sensory attributes in experimental pasta.

### 2.3. Prototype Scale-Up

Based on the preliminary results of SP sensory evaluation, all samples reported a good level of acceptance by panelists. Therefore, the prototype with 25% of SF (PSP25) and a control batch without SF (CP) were produced at the industrial level. However, due to the low score of acceptance of PSP25 dough by the industrial partner, an additional batch of PSP was made by adding half the SF (12.5%) and named PSP12. All prototypes (CP, PSP12 and PSP25) were produced, pasteurized and packaged in the shape of “trofie” pasta by Pastificio Martimucci S.r.l. (Altamura, Italy). The nutritional facts of SF and semolina are the same as those reported above (Section 2.2 and Supplementary Table S1). The kneading lasted 20 min. The system used (Pavan group, Padua, Italy) was Teflon. The pasta moisture content was 30%. The CP, PSP12 and PSP25 samples were heat-treated (73 °C; 3 min), packaged (500 g) in modified atmosphere and then thermally re-treated (73 °C; 3 min) and stored for 110 days at 4 °C.

### 2.4. Chemical Characterization of Pasta and Flours

The pH and total titratable acidity (TTA) were determined on uncooked pasta samples after homogenization of 10 g of pasta with 90 mL of distilled water for 3 min in a stomacher bag (Bag Mixer, Interscience International, Roubaix, France). The pH was determined by means of a pH-meter with a food-penetrating probe (Model 507, Crison, Milan, Italy). The TTA was expressed as the volume (mL) of 0.1 M NaOH solution required to reach a pH = 8.3. The activity water (A_w_) was measured in uncooked pasta, as well as in semolina and SF, at 25 °C using the Acqualab 4TE (Decagon Devices Inc., Pullman, WA, USA).

### 2.5. Centesimal Composition

Both prototypes (PSP12 and PSP25) and CP were analyzed for centesimal composition with the methods outlined in Appendix A.

### 2.6. Cooking Properties

According to the AACC66-50.01-approved method [25], aliquots (20 g) of each pasta sample were cooked in boiling tap water (200 mL) without salt to determine the optimal cooking time (OCT), weight loss during cooking (CL) and water absorption index (WAI).

Briefly, the disappearance of the whitish core of samples was evaluated every 30 s of cooking by crushing them between two transparent slides. The moment when the total disappearance of the nucleus was seen was taken as the OCT.

The solid matter lost in the cooking water was used to determine CL.

The WAI assessed the weight gain of pasta during cooking and was calculated according to the formula below:

WAI (%) = (cooked pasta weight − raw pasta weight)/(raw pasta weight) × 100

### 2.7. Color of Fresh Pasta

The color parameters of the dough were measured on sheets using a Chromameter CM-600d colorimeter (Konica Minolta Sensing, Osaka, Japan). The color indices, analyzed in triplicate (3 different spots of the surface area of each sample), were brightness (L*), red index (a*), and yellow index (b*). Color changes were also measured as total color difference (ΔE) indicating the chromaticity coordinates against the instrument’s blank, as previously detailed by Perri et al. [26].

### 2.8. In Vitro Starch Hydrolysis Index

The starch hydrolysis index (HI) was determined in vitro as previously described [27] and according to simulated starch digestion in vitro, as detailed elsewhere [28]. Free glucose was determined using the D-Fructose/D-Glucose Assay Kit (Megazyme, Wicklow, Ireland) and converted to hydrolyzed (digested) starch. The amount of starch digested at 3 h was expressed as the percentage of hydrolyzed starch out of the total determined after 16 h of incubation. To estimate the HI, white wheat bread (*Triticum aestivum*) was used as a control (HI = 100). As reported by [29], the predicted glycemic index (pGI) was calculated with the following equation:

pGI = 0.549 × HI + 39.71

### 2.9. Total Free Amino Acid (FAA) Analysis

The extract for total free amino acid (FAA) assessment was obtained from 1 g of crushed sample added with 4 mL of Tris-HCl 50 mM (pH 8.8) (ratio 1:4 *w*/*v*), left for 1 h at 4 °C to stir, and centrifuged (10,000 rpm, 20 min). Proteins and peptides were precipitated by adding 5% (*v*/*v*) solid-cold sulfur salicylic acid, keeping it at 4 °C for 1 h, and centrifuging at 15,000 rpm for 15 min. The supernatant was recovered and filtered through a non-sterile 0.22 μm filter and stored in a vial at −20 °C until use. Total and individual free amino acids from aqueous extracts were analyzed using an amino acid analyzer from the Biochrom 30 series (Biochrom Ltd., Cambridge Science Park, Cambridge, UK) with a cation exchange column (inner diameter of 20 cm × 0.46 cm). A mixture of amino acids of known concentration was used as standard. Amino acids were derivatized post-column with the nyhydrin reagent and detected by absorbance at 440 (proline and hydroxyproline) or 570 nm (all other amino acids) [30].

### 2.10. Antioxidant Activity

The antioxidant profile was assayed as the scavenging activity against the DPPH radical and considering the total polyphenol (TPC) and total flavonoid (TFC) content. Therefore, 1 g of cooked pasta was added to 10 mL of hydro-alcoholic solution (20:80 *v*/*v*), stirred (VM4 IDL agitator; 10 min, speed 6, at room temperature), treated in an ultrasonic bath for 15 min and then centrifuged (10,000 rpm, 10 min, 25 °C). The supernatant of hydroalcoholic extracts was harvested, whereas pellets underwent a second extraction step following the same procedure. The hydroalcoholic extracts were filtered through a nylon filter (porosity 0.45 mm, Sigma-Aldrich; Burlington, MA, USA).

Antioxidant activity was evaluated in terms of inhibition activity against the radical 2,2-Diphenyl-1-picrilidrazil (DPPH∙), as described by [31]. A blank (negative control) and a positive control containing 50 μL of ethanol–water solution or the synthetic antioxidant butylated hydroxytoluene (BHT) were included as a reference (1 g/L in ethanol–water solution), respectively, instead of sample. Absorbance was measured at 517 nm [32]. Total polyphenols (TPCs) were determined according to the Folin–Ciocalteu test [33]. In 20 μL of extract and 100 μL of Folin–Ciocalteu reagent, 980 μL of distilled water was added. After 3 min, 800 μL of Na_2_CO_3_ was added and then incubated for 1 h. Absorbance was read at 750 nm and the results have been expressed in μg of gallic acid equivalents (GAE) per gram of pasta. Total flavonoids (TFC) were determined by the colorimetric method of aluminum chloride (AlCl_3_) [34]. One aliquot (1 mL) of each extract was mixed with 0.2 mL of 10% AlCl_3_ methanol solution (*w*/*v*), 0.2 mL of sodium acetate (1 M), and 5.6 mL of distilled water. After incubation (in the dark, 30 min at room temperature), absorbance was measured with a spectrophotometer at 415 nm using an Agilent Cary 60 spectrophotometer (Cernusco, Milan, Italy). The results were derived from a quercetin calibration curve (0–200 μg/mL) prepared from a stock solution (5 mg/mL in methanol). The results have been expressed in mg quercetin equivalent (QE) per mL of extract.

### 2.11. In Vitro Pasta Digestion and Fecal Microbiota Profiling

Pasta prototypes (aliquots of 10 g added to 50 mL of distilled water and mixed in a stomacher for 2 min) were digested in vitro considering the enzymatic contribution of simulated oral (20 mg of α-amylase in 6.25 mL of CaCl_2_ (1 mM)), gastric (2.7 g of pepsin in 25 mL of 0.1 M HCl, pH 2) and intestinal (560 mg of pancreatin and 3.5 g of bile salts dissolved in 125 mL of 0.1 M NaHCO_3_, pH 7) fluids, following standardized procedures [35]. Thereafter, digested pasta samples were stored at −20 °C until the next use. The fecal medium was constituted as described previously [36]. To obtain the fecal microbiota inoculum, a fresh fecal sample (<1 h from delivery) from a healthy volunteer, a member of the research group who signed the informed consent and did not take drugs or probiotics in the last three months before the sample collection, was added at 32% (*w*/*v*) to saline solution (NaCl 0.9% *w*/*v*). In line with defined protocols [37], viable cells from fecal microbiota were added, and the final batch tube accounted for 7.5 mL of fecal medium suitably complemented with a volume of digest equal to 0.5 g of pasta and 2 mL of viable microbial suspension. Samples were incubated anaerobically (42 h, 37 °C) under stirring (100 ×g). Then, aliquots (5 mL) from fecal batches, mixed with 45 mL of sterilized saline solution (NaCl 0.9% *w*/*v*), were used to determine viable bacterial cells in PCA (total aerobes), Wilkins–Chalgren agar (total anaerobes), MRS agar (LAB), M17 agar (coccus-shaped LAB), VRBGA (coliforms), and *Bifidobacterium* agar (fecal bifidobacteria). Except for *Bifidobacterium* agar (Becton Dickinson; Le Pont de Claix, SA, France), other media were purchased from Oxoid Ltd. (Basingstoke, Hampshire, UK). Except for Wilkins–Chalgren agar and *Bifidobacterium* agar, the culture media were incubated aerobically. For the microbial growth, times and temperatures of incubation were provided by the manufacturer.

### 2.12. Gas Chromatography–Mass Spectrometry (GC-MS) Profiling of Fecal Batches

An aliquot equivalent to 1 g from each batch of fermented fecal matter was utilized to determine the profiles of volatile organic compounds (VOCs). The samples underwent a 10-min equilibration period at 60 °C. A solid-phase microextraction (SPME) fiber, coated with divinylbenzene/Carboxen/polydimethylsiloxane, was exposed to the samples for 40 min. VOCs were then thermally desorbed by promptly inserting the fiber into the heated injection port (220 °C) of a Clarus 680 gas chromatograph (Perkin Elmer, Beaconsfield, UK) equipped with an Rtx-WAX column (30 m × 0.25 mm i.d., 0.25 µm film thickness, Restek) and connected to a Clarus SQ8MS (PerkinElmer, Waltham, MA, USA). To serve as an internal standard, 10 µL of 4-methyl-2-pentanol (at a final concentration of 9.9 mg/L) was added. The resulting chromatograms were analyzed for peak identification using the National Institute of Standards and Technology (NIST) 2008 library, applying a peak area threshold of more than 1,000,000 and a match probability of 85% or higher, with the manual inspection of fragment patterns performed when necessary. Furthermore, the internal standard 4-methyl-2-pentanol was used to quantify the identified VOCs by interpolating their relative areas against the internal standard’s area.

### 2.13. Sensory Analysis

Ensuring accordance with The Code of Ethics of the World Medical Association (Declaration of Helsinki), the study received approval from the Local Ethics Committee IRCCS “Giovanni Paolo II” (study n. 1572, approved on 5 March 2024), and all subjects signed the informed consent. The sensory analysis was conducted by a panel of ten volunteers (5F:5M) of the research laboratory duly trained on the meaning of sensory attributes and the attribution of scores. Each type of pasta was identified by an alphanumeric code, cooked and served at room temperature, under natural lighting, in a random and non-repeating order. The attributes considered were color, odor, taste, bulkiness, adhesiveness, hardness and overall acceptability, using a 1 to 10 structured scale. The specific meaning of each attribute and the scale bar used to assign scores are listed in Appendix B.

### 2.14. Shelf-Life Assessment

To evaluate the shelf life of the PSP12 and PSP25 (and CP), microbiological analyses were carried out aimed at determining the cell densities of total aerobic mesophilic bacteria (UNI EN ISO 4833-1:2013 method), mesophilic lactic acid bacteria (LAB) (ISO 15214:1998), *Enterobacteriaceae* (ISO 21528-2:2017 method), enterococci (EN ISO 11133:2014), yeasts and molds (ISO 21527-2:2008 standard method), and coagulase-positive staphylococci (UNI EN ISO 6888-2:2004 method). As described previously [38], the aliquots (25 g) were aseptically taken from each packet and homogenized in 225 mL of Buffered Peptone Water (BPW) 0.1% in a stomacher bag (Bag Mixer, Interscience International, Roubaix, France) for 2 min. Decimal dilutions were plated onto the related culture media and incubated according to regulatory standards.

### 2.15. Statistics

All analyses were performed in triplicate. The data were subjected to univariate analysis of variance (ANOVA); the comparison between the means of the treatments was carried out by means of the Tukey procedure with a significance threshold set with values of *p* < 0.05 using the statistical software Statistica 12.5 (StatSoft Inc., Tulsa, OK, USA).

## 3. Results and Discussion

### 3.1. Chemical Characterization

The pH values were similar between the SF-enriched pasta samples, and both were slightly lower than that of CP (Table 1). The presence of organic acids, mainly oxalic and ascorbic with minor concentrations of citric and malic acids, in the spinach led to the lowering of pH values in SF-enriched pasta [39]. This reflected the TTA, which was higher in PSP25 compared to both CP and PSP12. The water activity (A_w_) values were the highest in CP and PSP25, whereas they decreased in PSP12 due to the lower values of A_w_ featuring the SF compared to semolina. Despite the observed variations, the A_w_ values detected in all pasta samples fall within the established range of acceptability (0.92–0.97) according to DPR No. 187/2001 (Decree of the Republic 9 February 2001, No. 187, art. 9 [40]).

### 3.2. Centesimal Composition

The results concerning the gross composition (Table 2) show that PSP25 had lower energy values and total carbohydrates than CP. By contrast, PSP25 reported the highest fiber values (11.3 g) as a result of the higher percentage of SF added into the formulation. As expected, PSP12 reported intermediate fiber values (5.2 g) that, according to EC Reg. 1924/2006 [19], allowed it to make the nutritional claim of being a “source of fiber”. With the growing awareness among consumers about the benefits of a balanced diet for health, there is increasing interest among producers in commercializing foods with health claims. Being not digestible by humans, both soluble and insoluble fibers play an important function related to intestinal motility [41]. Moreover, fiber, being bioavailable for the intestinal microbiota, can be fermented by health-promoting bacteria producing SCFAs, therefore providing a boosting effect on human health and supporting gastrointestinal homeostasis [42]. The total fats were lower in CP (1.7 g) than PSP25 (2.8 g) and PSP12 (2.3 g), whereas similar values of saturated fatty acids were found among samples. Additional results worthy of note included the higher content of vitamins B2, B3, B5 and B6, which were lower or absent in CP. Concerning minerals, SF-enriched pasta showed a significant increase in the contents of calcium, sodium, magnesium and potassium, in line with previous research that explored the conspicuous mineral content of this plant [43].

### 3.3. Cooking Properties

The OCT was determined for all prototypes (Table 3), with a slight increase observed in the dough without SF. The highest values of WAI were found in PSP25 (61.4), while CP had lower values (41.0). This is in line with the results of previous studies, which assessed that fiber-fortified pasta had a shorter cooking time [44] due to the considerable water absorption properties of dietary fiber [45]. The SI was lowest in CP (2.9) and highest in PSP25 (4.3) and PSP12 (4.1). Cooking loss (CL) refers to the dough’s resistance during cooking and is closely related to the strength of the protein network; good quality pasta should have a CL of no more than 7–8% [4]. In our study, CL ranged between 3.6 and 9% in CP and PSP25, because the breakdown of the protein matrix due to fibers, mainly insoluble ones, favors the leaching of starch during cooking, causing an increase in CL [46].

### 3.4. Color of Fresh Pasta

Color parameters of pasta samples before and after cooking were assessed (Table 4), revealing how increased percentages of SF significantly influenced the related values. Specifically, both raw and cooked PSP25 led to a significant decrease in brightness (L*) and yellow index (b*). The red index (a*) was also affected by the addition of SF, particularly in PSP25 reporting the highest values. While previous studies in this area of research have highlighted significant differences between results obtained from colorimeters and those from trained panelists in this area of research [47], our findings, summarized by ΔE* (which indicates the chromaticity coordinates against the instrument’s blank), allow us to conclude that although the percentages of SF significantly differentiated between pasta samples, cooking did not significantly affect the searched parameters.

### 3.5. In Vitro Starch Hydrolysis Index

**Figure 1:**
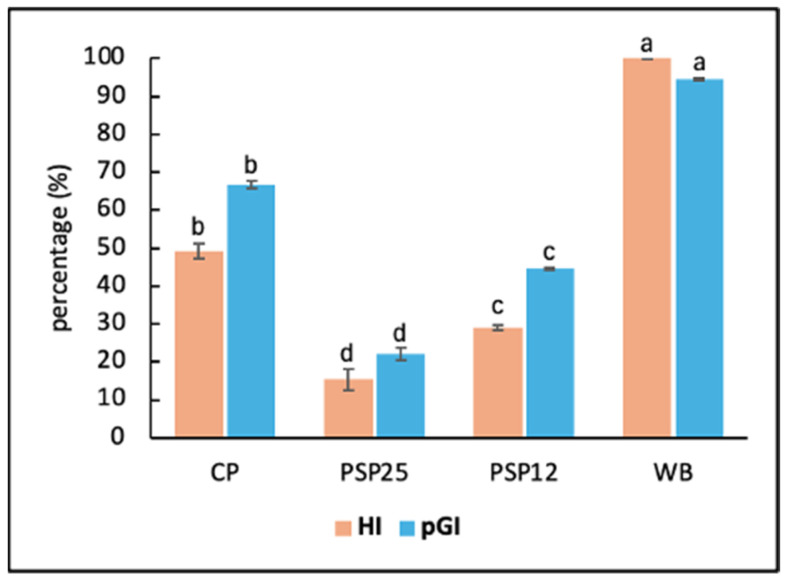
In vitro starch hydrolysis (HI) and predicted glycemic index (pGI) of cooked control pasta (CP) and enriched pasta with 25% and 12.5% SF (PSP25 and PSP12, respectively). White bread (WB) was used as the analytical positive control (HI = 100). On the stacked bars, different letters denote a significant *p*-value (*p* < 0.05; one-way ANOVA).

While the health importance of GI and glycemic load is debated, the global scientific community acknowledges that postprandial blood glucose is crucial for overall health, and GI is significant in preventing diabetes and obesity [48]. In pasta, dense starch and protein structures derived from extrusion processes allow for the slower digestion of starch, resulting in a low to medium GI [49]. Factors such as type of flour, temperature, humidity, drying and extrusion methods, and cooking time affect starch gelatinization and GI values. “Al dente” pasta typically has a low GI, but consuming large quantities in one meal can significantly impact blood sugar levels and lead to high calorie intake [50]. Recent research has focused on creating low-GI foods using plant-based ingredients in fresh pasta production [46]. In our study, we analyzed cooked pasta samples (CP, PSP12, and PSP25) for in vitro starch hydrolysis. Incorporating SF significantly impacted the HI and the related pGI because the enriched samples PSP12 and PSP25 had lower (*p* < 0.05) HI and pGI values than CP (Figure 1).

### 3.6. Total Free Amino Acid Analysis

**Figure 2:**
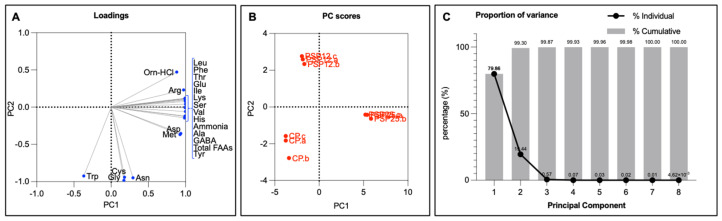
Principal component analysis (PCA) of total free amino acids (FAAs) found in cooked control pasta (CP) and enriched pasta with 25% and 12.5% SF (PSP25 and PSP12, respectively). (**A**) Loading of variables (FAAs); (**B**) PC score of samples; (**C**) PC contribution to total variance.

The enrichment of pasta with SF clearly influenced the amino acid composition of samples, with SF significantly impacting the concentration of free amino acids (FAAs) in cooked products. The related principal component analysis (PCA) explained 99.3% of the total variance based on the cumulative contribution of PC1 (79.86%) and PC2 (19.44%), which, respectively, described differences occurring in PSP25 and PSP12 (Figure 2) from CP. According to the positive score of PC1, the highest concentrations of most of the total FAAs (Supplementary Table S2) caused the differentiation of PSP25 from other samples (Figure 2A). Although the health benefits of spinach are mostly associated with the notable intake of vitamins (i.e., K, A, C and folate) that partially meet or exceed their respective Recommended Dietary Allowance [23], its intake also provides around 20% of the entire spectrum of all EAAs, with slight variations in percentage depending on the compound. Moreover, the gamma-aminobutyric acid (GABA) concentration was around 300 mg in both PSP12 and PSP25, the same value that various institutions have recognized as the beneficial dose for this compound [51]. GABA acts as a tranquilizer, effectively reducing stress and anxiety and regulating nerve impulses, as well as breaking down body fats, aiding child development, and controlling high blood pressure [51]. The lower concentrations of cysteine (Cys), glycine (Gly) and asparagine (Asn), and the highest concentration of tryptophan (Trp), led instead to the significant differentiation of PSP12 from both PSP25 and CP.

### 3.7. Antioxidant Activity

**Figure 3:**
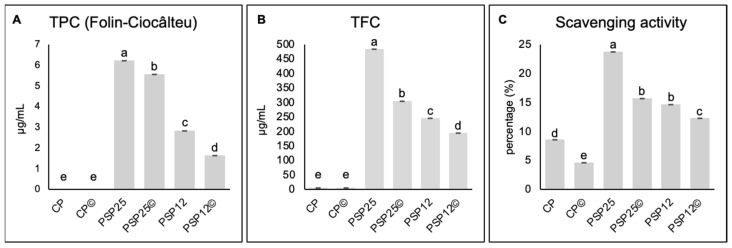
Total phenols (**A**), total flavonoids (**B**), and scavenging activity (**C**) assays of uncooked (without marks) or cooked (©) control pasta (CP) and enriched pasta with 25% and 12.5% of spinach flour (PSP25 and PSP12, respectively). On the stacked bars, different letters dentote a significant difference (*p* < 0.05; one-way ANOVA).

Both the TPC and TFC of spinach-enriched pasta (PSP25 and PSP12) were higher than those of CP (Figure 3A,B). Notably, although cooking significantly reduced these values, as also demonstrated by previous authors [52], the residual concentrations maintained their relative proportions (and significance) in comparison. This was further confirmed by the scavenging activity assay (Figure 3C), which showed the highest value for uncooked PSP25 and the lowest for CP, consistent with previous studies highlighting the significant potential of spinach to prevent oxidative stress-related issues as the result of the considerable amounts of oxalic and ascorbic acids usually contained in spinach leaves [53,54].

### 3.8. In Vitro Pasta Digestion and Fecal Microbiota Profiling

By simulating colon fermentation, the efficacy of digested fortified pasta samples in influencing fecal microbiota cell viability was tested in batches fermented for 20 and 42 h. No clear trends emerged from the analysis of the data. At T20, the samples showed increased viability of total bacteria (TB), *Enterobacteriaceae*, and LAB. In comparison, the viability of lactic streptococci, clostridial cells, and bifidobacteria was increased at T42. An exception can be made for bacillus-shaped LAB in PSP25 compared against both PSP12 and CP, as their ability to metabolize a great amount of fiber [55] meant that the differences in the values of microbial cell density did not reach the significance threshold.

### 3.9. Volatile Organic Compound (VOC) Profiling of Fecal Batches

**Figure 4:**
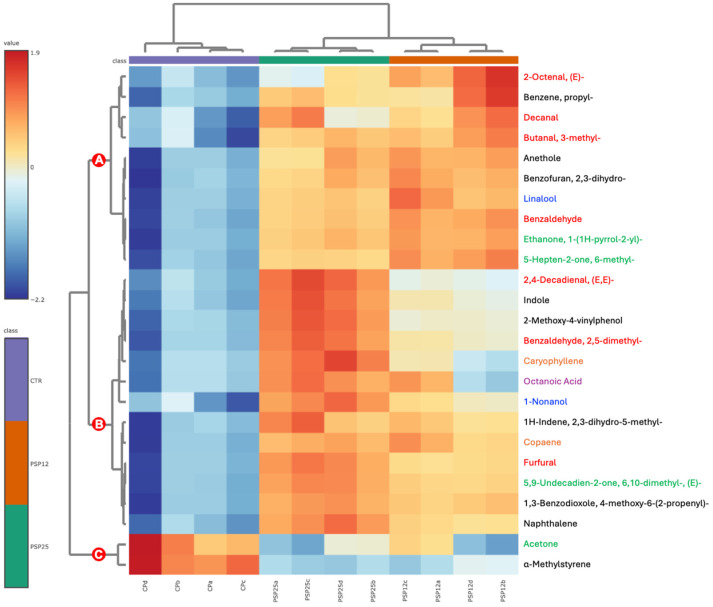
According to the top 25 volatile organic compounds based on Partial Least Squares Discriminant Analysis (PLSDA), a heatmap is shown with the clustering of cooked control pasta (CP) and enriched pasta with 25% and 12.5% of SF (PSP25 and PSP12, respectively) digested in vitro and fermented by fecal microbiota. Different font colors were used to identify VOCs with the related chemical classes: aldehydes (red), alcohols (blue), carboxylic acids and derivatives (purple), aromatic compounds (black), ketones (green), terpenes (orange).

The GC-MS profiling of fecal batches identified 64 different peaks of volatile organic compounds (VOCs) (Supplementary Table S3). Among these, three VOCs (butane, 2,2-dimethyl-; hexane, 2,3,4-trimethyl-; hexane, 3,3-dimethyl-) were found exclusively in CP, likely due to the absence of spinach fibers. Nine VOCs were detected in PSP25 but not in PSP12, possibly because their concentrations were below the detection limit in PSP12. All 64 VOCs were processed using Partial Least Squares Discriminant Analysis (PLS-DA) to identify the key contributors for discrimination between samples. A heatmap with clustering was then created, revealing that three clusters led to a clear separation of samples according to the type of pasta fermented by fecal microbiota (Figure 4). Cluster A included 10 of the top 25 VOCs and was the most representative of PSP25 samples, whereas PSP12 samples showed intermediate values. Cluster B comprised 13 VOCs predominantly found in PSP25 samples. Cluster C, which included only two VOCs, highlighted the differences between SF-enriched samples and CP. Specifically, these two compounds were α-methylstyrene and acetone, resulting from different metabolic pathways—α-methylstyrene from the breakdown of aromatic amino acids like phenylalanine [56], and acetone from various pathways such as acetone–butanol–ethanol (ABE) fermentation, acetoacetate metabolism, fatty acid β-oxidation, and ketogenesis. These metabolites and their associated pathways suggest a higher involvement of different bacterial species, such as *Clostridium*, *Bacteroides*, and certain members of the *Enterobacteriaceae* family—taxa known to act as pathobionts in the human gut environment [57].

### 3.10. Sensory Analysis

**Figure 5:**
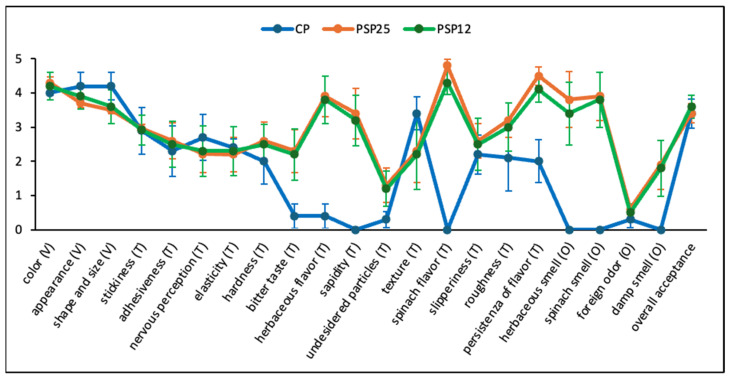
Sensory evaluation for n.3 visual (V), n.14 taste (T) and n.4 olfactory (O) attributes of control pasta (CP) and enriched pasta with 25% and 12.5% SF (PSP25 and PSP12, respectively). In the figure, the last descriptor refers to the overall acceptance of the pasta samples. Scores ranged from 0 (poor) to 5 (excellent).

Visual (V), taste (T) and olfactory (O) analyses were carried out to reveal differences between the CP and the spinach-enriched pasta (Figure 5). The CP scored slightly higher in terms of appearance, shape, and size. However, the pasta with SF exhibited more particles that were difficult to swallow and had a perceived bitter taste. Minor, but not non-significant (*p* > 0.05), differences were observed by panelists in the texture-related taste attribute. This highlights a limitation of the present study, as the absence of a focused, instrumental texture analysis prevented a more detailed assessment of differences between samples, which help could better determine if a significant difference exists. Despite these issues, the overall acceptability was higher for PSP12 compared to CP (3.6 and 3.4, respectively), although the values were quite similar. In conclusion, the findings demonstrate that incorporating SF into pasta introduces herbaceous smells and flavors, which positively influence the overall acceptability of the product. This suggests that producing and consuming pasta enriched with vegetables or vegetable-based flours can not only enhance the nutritional profile, but also improve the flavor, as also supported by previous research [58]. However, a potential challenge is overcoming cultural resistance to purchasing and consuming innovative products like pasta enriched with vegetable-based flours, which deviate from the traditional characteristics of the product.

### 3.11. Shelf-Life Assessment

The microbiological characterization (Table 5) was carried out throughout the sample’s shelf-life, determining the microbiological cell density at 0, 30, 60, 90, and 110 days in both SF-containing pasta (PSP12 and PSP25) and CP stored at 4 °C. The cell density of coagulase-positive staphylococci was less than 3 log CFU/g in all samples. Coliform densities (not reported) and *Enterobacteriaceae* were less than 2 log CFU/g in all samples up to 110 days. In PSP25, the TMA was approximately 5 log CFU/g at the beginning and 4.75 log CFU/g after 110 days of storage. In PSP12, the TMA was 4.4 log CFU/g at T0 and remained almost at the same density until the end of storage. In CP, the TMA was approximately 3 log CFU/g at the beginning of storage, increasing up to 4.7 log CFU/g at T110. The density of LAB in PS25 was approximately 3 log CFU/g at the beginning of refrigerated storage, with no significant differences at T110. The densities of yeasts and molds were approximately 4 log CFU/g in PSP25 and PSP12 at the end of storage, which is slightly higher than in the CP. In CP, yeasts and molds grew to 3 and 2 log CFU/g, respectively, after 110 days of storage. Overall, all the values of the microbiological indicators are within the desired range, respecting the microbiological limits for fresh pasta set out in EC Reg. 852/2004 [59] and EC Reg. 1441/2007 [60].

## 4. Conclusions

Substituting semolina with 12.5% *w*/*w* of SF in fresh pasta production yielded a product with acceptable cooking performance, texture, and quality. This was attributed to the high protein content of SF (rich in cysteine) and the moderate reduction in gluten due to the semolina substitution. The substitution allowed the pasta produced to be classified as a “source of fiber” at 12.5% replacement (PSP12) and a “high fiber content” product at 25% (PSP25) replacement. The PSP12 reported good properties and shelf-life during refrigerated storage, making it viable for industrial production with potential nutritional benefits. By consuming 100 grams of PSP12, it is possible to derive approximately 30% of the daily requirement for vitamin B2, 50% of the requirement for vitamin B5 and 1.5–4 mg of the requirement for iron, and to fully meet the magnesium requirement (with 800 mg/kg of magnesium detected in the product). Given that this study supports the nutritional benefits of fortified foods and encourages innovation in pasta production, future in vivo studies should be conducted to evaluate and validate the long-term health benefits of consuming this pasta prototype.

## Figures and Tables

**Table 1:** pH, TTA and activity water of control pasta (CP) and enriched pasta with 25% and 12.5% of SF (PSP25 and PSP12, respectively). Of the used ingredients (semolina and spinach-flour), the A_w_ was also assessed.

Sample	pH	TTA	A_w_
CP	6.55 ± 0.33 ^a^	1.0 ± 0.05 ^b^	0.95 ± 0.05 ^a^
PSP25	6.24 ±0.31 ^b^	2.3 ± 0.12 ^a^	0.92 ± 0.05 ^b^
PSP12	6.28 ±0.31 ^b^	1.3 ± 0.07 ^b^	0.93 ± 0.05 ^b^
Semolina	-	-	0.54 ± 0.03 ^c^
SF	-	-	0.42 ± 0.02 ^d^

Within the same column, different superscript letters show a significant *p*-value (*p* < 0.05).

**Table 2:** Nutritional facts of control pasta (CP) and enriched pasta with 25% and 12.5% of SF (PSP25 and PSP12, respectively).

Nutritional Facts	CP	PSP25	PSP12
Moisture (g/100 g)	27.3	31.2	29
Energy value (Kcal/100 g)	294	254	273
Energy value (KJ/100 g)	1248	1070	1158
Carbohydrates (g/100 g)	57.6	38.8	48.5
of which sugars	1.17	1.29	1.21
Fats (g/100 g)	1.7	2.8	2.3
of which saturated	0.3	0.4	0.4
of which monounsaturated	0.35	2	1.2
of which polyunsaturated	1.02	0.4	0.7
Proteins (% N × 6.25)	11.4	12.8	12.1
Fiber (g/100 g) *	1.5	11.3	5.2
of which insoluble	0.6	5.7	2.6
of which soluble	0.9	5.6	2.6
(1-3) (1-4) Beta-glucans	n.d.	0.205	0.101
Salt (g/100 g)	0.001	0.24	0.11
Ashes (g/100 g)	0.79	4.59	2.75
**Vitamins (µg/kg)**			
Vitamin B1	540	1240	583
Vitamin B2	40	642	317
Vitamin B3	n.d. **	549	221
Vitamin B5	n.d. **	6960	3250
Vitamin B6	n.d. **	3.9	2
**Minerals (mg/kg)**			
Ca	141	3590	1815
Na	2	963	430
Zn	618	416	103
Fe	n.d. *	63	24
Cd	0.04	n.d. **	n.d. **
Pb	n.d. *	n.d. **	n.d. **
Mg	264	1334	599
K	140	1157	629

n.d., not detected; * fibers include the cumulative assessment of both dietary and crude fiber; ** below the detection threshold 10 µg/kg.

**Table 3:** Cooking properties (optimal cooking time, OCT; water absorption index, WAI; swelling index, SI and cooking loss, CL) of control pasta (CP) and enriched pasta with 25% and 12.5% of spinach flour (PSP25 and PSP12, respectively).

Sample	OCT (min)	WAI (g/100 g)	SI	CL (g/100 g)
CP	10	41.0 ± 2.0 ^c^	2.9 ± 0.1 ^b^	3.6 ± 0.2 ^c^
PSP25	8	61.4 ± 3.1 ^a^	4.3 ± 0.2 ^a^	9.0 ± 0.5 ^a^
PSP12	9	51.9 ± 2.6 ^b^	4.1 ± 0.2 ^a^	6.6 ± 0.3 ^b^

Within the same column, different superscript letters show a significant *p*-value (*p* < 0.05).

**Table 4:** Colorimetric profiling of uncooked (U) or cooked (C) control pasta (CP) and enriched pasta with 25% and 12.5% of spinach flour (PSP25 and PSP12, respectively).

**Sample**	L*	a*	b*	ΔE*
CP (U)	77.73 ± 1.18 ^a^	−7.03 ± 0.15 ^a^	27.82 ± 0.46 ^a^	23.78 ± 0.60 ^c^
CP (C)	77.98 ± 2.96 ^a^	−6.32 ± 0.54 ^ab^	22.05 ± 0.55 ^b^	20.20 ± 2.58 ^c^
PSP25 (U)	26.51 ± 0.54 ^c^	−4.56 ± 0.14 ^c^	10.18 ± 0.33 ^e^	67.11 ± 0.78 ^a^
PSP25 (C)	27.22 ± 0.45 ^c^	−4.39 ± 0.19 ^c^	10.66 ± 0.32 ^e^	66.14 ± 0.46 ^a^
PSP12 (U)	32.83 ± 0.85 ^b^	−6.15 ± 0.38 ^b^	13.12 ± 0.83 ^d^	60.74 ± 0.80 ^b^
PSP12 (C)	34.63 ± 3.49 ^b^	−6.25 ± 1.22 ^ab^	15.41 ± 2.26 ^c^	59.10 ± 3.19 ^b^

Within the same column, different superscript letters show a significant *p*-value (*p* < 0.05; one-way ANOVA).

**Table 5:** Shelf-life assessment of pasta samples (CP, PSP25 and PSP12) at 0, 30, 60, 90 and 110 days according to viable microbial counts of total mesophilic aerobes (TMA), lactic acid bacteria (LAB), Staphylococcus (*Staph*.), *Enterobacteriaceae* (*Enterob*.), yeasts and molds.

Storage	Sample	TMA	LAB	*Staph*.	*Enterob.*	Yeasts	Molds
T0	CP	3.3 ± 0.8	1.6 ± 0	n.d.	2.3 ± 0	<1	<2
	PSP25	5.0 ± 0.1	3.3 ± 0.1	3.1 ± 0.1	2.7 ± 0.1	2.5 ± 0.2	3.7 ± 0.4
	PSP12	4.4 ± 0.1	2.9 ± 0.2	<2	2.4 ± 0.1	<2	3.0 ± 0.1
T30	CP	3.0 ± 0.1	2.3 ± 0.4	n.d.	<2	<1	<2
	PSP25	5.1 ± 0.1	3.8 ± 0.1	2.8 ± 0.1	<2	2.4 ± 0.1	3.5 ± 0.5
	PSP12	4.2 ± 0.2	3.2 ± 0.2	2.2 ± 0.2	<2	<2	3.0 ± 0.2
T60	CP	3.6 ± 0.1	2.2 ± 0.2	n.d.	<2	3.0 ± 0.1	2.2 ± 0.6
	PSP25	4.0 ± 0.1	3.9 ± 0.1	<2	<2	4.4 ± 0.1	3.1 ± 0.4
	PSP12	4.4 ± 0.1	3.1 ± 0.3	<2	<2	4.3 ± 0.2	3.1 ± 0.1
T90	CP	4.6 ± 0.1	2.3 ± 0.3	2.9 ± 0.2	<2	3.4 ± 0.1	2.1 ± 0.2
	PSP25	4.1 ± 0.1	3.6 ± 0.1	2.2 ± 0.1	<2	4.4 ± 0.1	4.4 ± 0.2
	PSP12	5.0 ± 0.1	3.3 ± 0.2	2.5 ± 0.3	<2	4.3 ± 0.2	4.2 ± 0.1
T110	CP	4.7 ± 0.1	2.4 ± 0.2	2.2 ± 0.2	<2	3.3 ± 0.1	2.0 ± 0.1
	PSP25	4.4 ± 0.1	3.1 ± 0.1	2.1 ± 0.1	<2	4.4 ± 0.1	4.0 ± 0.3
	PSP12	4.8 ± 0.1	3.9 ± 0.3	2.4 ± 0.1	<2	4.3 ± 0.2	4.0 ± 0.1

n.d., not detected.

## Data Availability

The data presented in this study are available on request from the corresponding authors.

## Supplementary Materials

The following supporting information can be downloaded at: https://www.mdpi.com/article/10.3390/foods13223608/s1. Table S1. Nutritional facts on 100 g of spinach flour; Table S2. Concentration (mg/kg) of total free amino acids (FAAs) found in cooked control pasta (CP) and enriched pasta with 25% and 12.5% of spinach-flour (PSP25 and PSP12, respectively). Table S3. Relative concentration (mg/Kg) of volatile organic compounds (VOCs) found in fecal batches that fermented control pasta (CP) and enriched pasta with 25% and 12.5% of spinach-flour (PSP25 and PSP12, respectively).

## Appendix A

For the for centesimal composition, the parameters considered were moisture (Method UNI EN ISO 712:2010); energy value (Method Reg. CE 1169/2011GU CE L.304 del 22-11-2011-All. XIV); total carbohydrates (Method L-MI014 rev.0 2016 LOQ: 0.02); total fats (Method DM 23-07-94 SO n°4 G.U. n°186 del 10-08-1994); saturated fatty acids (Method AOAC 996.06); monounsaturated and polyunsaturated fatty acids (Method from calculation after determination of methyl esters according to EEC Reg. 2568/1991 and subsequent amendments); total proteins (Method UNI CEN ISO/TS 16634-2:2016 LOQ: 0.35); total dietary fiber (Method AOAC 985.29 LOQ: 0.1), insoluble and soluble fiber (Method AOAC 993.19:1996 LOQ: 0.1); beta-glucan (Method AOAC 995.16 2005); salt (from calculation after determination of sodium according to L-MI068 LOQ: 0.001); ash (Method ISO 2171:2007); vitamins B1 (Thiamine) (Method L-MI094 rev.0 2022 LOQ: 0.1), B2 (Riboflavin) (Method L-MI094 rev.0 2022 LOQ: 0.1), B3 (Niacinamide) (Method L-MI094 rev.0 2022 LOQ: 0.1), B5 (Pantothenic Acid) (Method L-MI094 rev.0 2022 LOQ: 0.1) and B6 (Pyridoxal, Pyridoxamine, Pyridoxine) (Method L-MI094 rev.0 2022 LOQ: 0.1); minerals (calcium, sodium, zinc, iron, magnesium, potassium) (Met: L-MI068 rev.0 2020LOQ: 10); and heavy metals (cadmium, lead) (Method L-MI068 rev.0 2020 LOQ: 0.01).

## Appendix B

For the sensory evaluation, the visual attributes considered were the intensity of the color, the appearance, the shape and the size. For the taste examination, the attributes considered were stickiness, adhesiveness, nerve, elasticity, hardness, bitter taste, herbaceous taste, flavor, presence of undesired particles, texture, spinach taste, slipperiness and roughness. The olfactory examination considered as descriptors herbaceous odor, spinach odor, duly odor and foreign odor. The score for each sensory attribute ranged from 0 (worst) to 5 (excellent).
