# Supplementary material for: Nutritional, Biochemical, and Functional Properties of Spinach Leaf-Enriched Dough: A Healthier Alternative to Conventional Pasta
Source: Foods. 2024 Nov 12;13(22):3608. doi: 10.3390/foods13223608 (PMC11593830; doi:10.3390/foods13223608)
Supplement: Supplementary file 1 [file foods-13-03608-s001.zip › foods-3298602-supplementary.pdf]

Supplementary Table S1: Nutritional facts on 100g of spinach flour.

Nutritional facts of *Spinacia oleracea* flour (SF).	
**Moisture (%)**	< 8
Particle size (µm)	< 200
Sodium chloride (mg)	732 (±200)
Fibre (g)	22.50 (±5.05%)
Energy (Kcal)	278
Energy (KJg)	1161
Carbohydrates (g)	7.24 (±15.05%)
of which sugars (g)	5.5
Total fats (g)	3.75 (±2.05%)
of which saturated (g)	0.45
Proteins (g)	35 (±10.05%)

Supplementary Table S2: Concentration (mg/Kg) of total free amino acids (FAAs) found in cooked control pasta (CP) and enriched pasta with 25% and 12.5% of SF (PSP25 and PSP12, respectively).

Amino acid (mg/kg)	CP	PSP25	PSP12
Aspartic acid	182.4 ±3.6[c]	262.6 ±5.3[a]	205.1 ±3.3[b]
Threonine	15.6 ±0.3[c]	81.8 ±1.6[a]	36.3 ±0.7[b]
Serine	22.3 ±0.4[c]	95.6 ±1.9[a]	43.0 ±0.9[b]
Asparagine	341.4 ±6.8[a]	336.1 ±6.7[a]	333.3 ±4.1[a]
Glutamic Acid	147.2 ±2.9[c]	314.6 ±6.3[a]	195.2 ±3.9[b]
Glycine	28.9 ±0.6[a]	27.0 ±0.5[a]	27.4 ±0.3[a]
Alanine	81.7 ±1.6[c]	220.0 ±4.4[a]	101.1 ±2.0[b]
Valine	21.6 ±0.4[c]	171.8 ±3.4[a]	56.3 ±1.1[b]
Cysteine	25.4 ±0.5[a]	25.2 ±0.5[a]	22.0 ±0.4[b]
Methionine	6.4 ±0.1[b]	12.0 ±0.2[a]	5.6 ±0.1[c]
Isoleucine	36.7 ±0.7[c]	91.6 ±1.8[a]	51.0 ±1.0[b]
Leucine	25.1 ±0.5[c]	133.3 ±2.7[a]	59.2 ±1.2[b]
Tyrosine	19.4 ±0.4[c]	52.5 ±1.0[a]	21.2 ±0.4[b]
Phenylalanine	19.1 ±0.4[c]	75.5 ±1.5[a]	36.2 ±0.7[b]
GABA	113.8 ±2.3[c]	289.4 ±5.8[a]	127.4 ±2.5[b]
Ammonia	20.0 ±0.4[c]	43.5 ±0.9[a]	25.5 ±0.5[b]
Ornithine-HCl	n.d.*	5.0 ±0.1[a]	3.2 ±0.1[b]
Lysine	22.1 ±0.4[c]	67.3 ±1.3[a]	35.8 ±0.7[b]
Histidine	11.2 ±0.2[c]	28.2 ±0.6[a]	14.6 ±0.3[b]
Tryptophan	135.7 ±2.7[a]	90.7 ±1.8[b]	98.4 ±1.4[b]
Arginine	47.8 ±1.0[c]	248.7 ±5.0[a]	130.6 ±2.6[b]
Total proteins	1323.7 ±26.5[a]	2672.2 ±53.4[a]	1416.3 ±28.3[b]

Supplementary Table S3: Relative concentration (mg/Kg) of volatile organic compounds (VOCs) found in fecal batches that fermented control pasta (CP) and enriched pasta with 25% and 12.5% of spinach-flour (PSP25 and PSP12, respectively).

**Compound**	**CP**	**PSP12**	**PSP25**
alpha-Methylstyrene	0.03 ±0.00	0.01 ±0.01	n.d.*
1-Butanol, 3-methyl-	n.d.	0.01 ±0.01	0.00 ±0.012
1-Decene	n.d.	n.d.	0.01 ±0.01
1-Dodecanol	n.d.	n.d.	0.00 ±0.01
1-Hexanol, 2-ethyl-	0.04 ±0.02	n.d.	0.05 ±0.02
1-Hexanol, 4-methyl-	n.d.	0.01 ±0.00	0.01 ±0.01
1-Nonanol	0.03 ±0.01	0.04 ±0.00	0.05 ±0.00
1-Octanol	n.d.	n.d.	0.02 ±0.02
1-Pentanol	0.01 ±0.00	0.05 ±0.01	0.01 ±0.00
1,3-Benzodioxole, 4-methoxy-6-(2-propenyl)-	n.d.	0.12 ±0.00	0.15 ±0.02
1H-Indene,2,3-dihydro-5-methyl-	n.d.	0.06 ±0.01	0.08 ±0.02
2-Decanol	0.01 ±0.01	0.01 ±0.00	n.d.
2-Methoxy-4-vinylphenol	n.d.	0.02 ±0.00	0.04 ±0.00
2-Naphthalenol	n.d.	n.d.	0.01 ±0.00
2-Nonenal, (E)-	0.36 ±0.15	0.48 ±0.01	0.39 ±0.03
2-Octenal, (E)-	0.09 ±0.04	0.27 ±0.03	0.17 ±0.03
2-Undecene, 6-methyl-, (Z)-	0.01 ±0.01	n.d.	0.00 ±0.01
2,4-Decadienal, (E,E)-	0.03 ±0.02	0.07 ±0.00	0.16 ±0.01
2,4-Di-tert-butylphenol	0.01 ±0.01	0.05 ±0.00	0.02 ±0.00
2(3H)-Furanone, 5-hexyldihydro-	0.03 ±0.02	0.01 ±0.00	0.03 ±0.00
5-Hepten-2-one, 6-methyl-	0.02 ±0.01	0.18 ±0.01	0.13 ±0.00
5,9-Undecadien-2-one, 6,10-dimethyl-, (E)-	0.04 ±0.03	1.66 ±0.10	2.36 ±0.18
Acetone	0.10 ±0.01	0.07 ±0.02	0.06 ±0.01
Acetophenone	0.01 ±0.01	0.03 ±0.00	0.02 ±0.01
Anethole	n.d.	0.03 ±0.00	0.02 ±0.01
Benzaldehyde	0.04 ±0.01	0.42 ±0.03	0.34 ±0.03
Benzaldehyde, 2,5-dimethyl-	n.d.	0.07 ±0.01	0.16 ±0.01
Benzene, 1-methyl-2-(1-methylethyl)-	0.00 ±0.01	0.01 ±0.02	n.d.
Benzene, 1-methyl-3-(1-methylethyl)-	0.01 ±0.00	0.01 ±0.02	n.d.
Benzene, propyl-	0.00 ±0.01	0.10 ±0.05	0.08 ±0.01
Benzofuran, 2,3-dihydro-	n.d.	0.02 ±0.00	0.02 ±0.00
Benzyl Alcohol	n.d.	n.d.	0.01 ±0.00
Butanal, 3-methyl-	0.02 ±0.02	0.06 ±0.00	0.05 ±0.01
Butane, 2,2-dimethyl-	0.01 ±0.02	n.d.	n.d.
Butylated Hydroxytoluene	0.01 ±0.01	0.01 ±0.01	0.02 ±0.01
Caryophyllene	n.d.	0.01 ±0.01	0.03 ±0.01
Copaene	n.d.	0.03 ±0.01	0.03 ±0.00
Decanal	0.18 ±0.09	0.35 ±0.05	0.32 ±0.08
Dodecane	0.02 ±0.00	0.02 ±0.02	0.03 ±0.01
Ethanol	n.d.	n.d.	0.01 ±0.00
Ethanol, 2-phenoxy-	0.01 ±0.02	n.d.	0.01 ±0.00
Ethanone, 1-(1-cyclohexen-1-yl)-	n.d.	0.01 ±0.00	0.09 ±0.11
Ethanone, 1-(1H-pyrrol-2-yl)-	n.d.	0.03 ±0.00	0.03 ±0.00
Furan, 2-pentyl-	0.21 ±0.06	0.09 ±0.03	0.35 ±0.06
Furfural	n.d.	0.01 ±0.00	0.01 ±0.00
Hexadecane	0.01 ±0.02	n.d.	0.02 ±0.01
Hexadecanoic acid, methyl ester	n.d.	n.d.	0.01 ±0.00
Hexanal	0.06 ±0.03	0.08 ±0.00	0.04 ±0.02

Hexane, 2,3,4-trimethyl-	0.01 ±0.01	n.d.	n.d.
Hexane, 3,3-dimethyl-	0.01 ±0.01	n.d.	n.d.
Hexanoic acid	0.01 ±0.00	0.03 ±0.00	0.01 ±0.02
Indole	n.d.	0.02 ±0.00	0.04 ±0.00
Limonene	0.02 ±0.02	0.06 ±0.04	0.01 ±0.01
Linalool	n.d.	0.02 ±0.01	0.02 ±0.00
Naphthalene	0.01 ±0.00	0.03 ±0.00	0.04 ±0.01
Nonanal	0.23 ±0.33	0.67 ±0.35	0.01 ±0.01
Octanoic Acid	n.d.	0.02 ±0.02	0.04 ±0.00
Pentanoic acid	0.01 ±0.00	0.01 ±0.02	0.01 ±0.02
Phenol, 4-(1,1-dimethylpropyl)-	0.01 ±0.00	0.01 ±0.00	0.03 ±0.00
Phenylethyl Alcohol	n.d.	0.02 ±0.00	n.d.
Styrene	0.03 ±0.01	0.02 ±0.00	0.03 ±0.01
Tetradecane	0.07 ±0.02	0.02 ±0.01	0.04 ±0.00
Tridecane	0.08 ±0.03	0.10 ±0.02	0.06 ±0.00
Undecane, 4,7-dimethyl-	n.d.	0.01 ±0.01	n.d.

(*) n.d.: not detected; lower than the detection threshold.
